# Efficacy and Safety of Baxdrostat for Hypertension: A Systematic Review and Meta‐Analysis of Three Phase 2/3 Randomized Controlled Trials

**DOI:** 10.1002/clc.70391

**Published:** 2026-06-23

**Authors:** Rahul Falodia, Ehsanullah Alokozay, Mohammad Idrees, Darsh Paghdar, Ali Msheik, Taha Alam, Saeedullah Zadran

**Affiliations:** ^1^ All India Institute of Medical Sciences Jodhpur India; ^2^ Faculty of Medicine Kandahar University Kandahar Afghanistan; ^3^ Saidu Medical College Saidu Sharif Swat Pakistan; ^4^ Neurosurgery Department, Neuroscience Institute Hamad Medical Corporation Doha Qatar; ^5^ Dow University of Health Sciences Karachi Pakistan; ^6^ White River Medical Center Batesville Arkansas USA

**Keywords:** aldosterone synthase inhibitor, ambulatory blood pressure, baxdrostat, CYP11B2, hyperkalemia, meta‐analysis, resistant hypertension, systematic review

## Abstract

**Background:**

Baxdrostat is a selective aldosterone synthase (CYP11B2) inhibitor for treatment‐resistant and uncontrolled hypertension, evaluated in phase 2/3 RCTs.

**Objectives:**

To estimate pooled placebo‐corrected reductions in seated office and ambulatory SBP, characterize the dose–response relationship, assess cortisol selectivity, and quantify hyperkalemia risk.

**Methods:**

We conducted a systematic review and meta‐analysis of phase 2/3 randomized, double‐blind, placebo‐controlled trials of baxdrostat in hypertension, searching MEDLINE/PubMed, EMBASE, ClinicalTrials.gov, and NEJM.org to March 2026. The primary outcome was placebo‐corrected change in seated office SBP, analyzed using random‐effects meta‐analysis (REML) with metafor in R. Risk of bias and certainty were evaluated using Cochrane RoB 2.0 and GRADE, respectively.

**Results:**

Three RCTs (BrigHTN, BaxHTN, Bax24; *N* = 1285) were included. The pooled seated SBP reduction was −9.44 mmHg (95% CI: −11.09 to −7.79; *I* ^2^ = 0.0%), with dose subgroup estimates of −8.57 mmHg (1 mg) and −10.11 mmHg (2 mg) and a clear dose–response in BrigHTN (slope −4.2 mmHg/mg; *R* ^2^ = 0.96). Bax24 demonstrated a 24 h ambulatory SBP reduction of −14.0 mmHg (95% CI: −17.2 to −10.8). Serum cortisol was unsuppressed at all doses, while the pooled OR for potassium ≥ 6.0 mmol/L was 5.14 (95% CI: 1.76 to 14.97). GRADE certainty was moderate for seated SBP, low to moderate for ambulatory outcomes, and low for hyperkalemia.

**Conclusions:**

Baxdrostat produces meaningful, selective BP reductions across hypertensive populations. Hyperkalemia is manageable with structured monitoring. Certainty is limited by a few trials, a short follow‐up, and safety imprecision. Long‐term cardiovascular outcome data are needed before guideline incorporation.

## Graphical abstract

Baxdrostat: Effective blood pressure reduction with preserved cortisol levels.

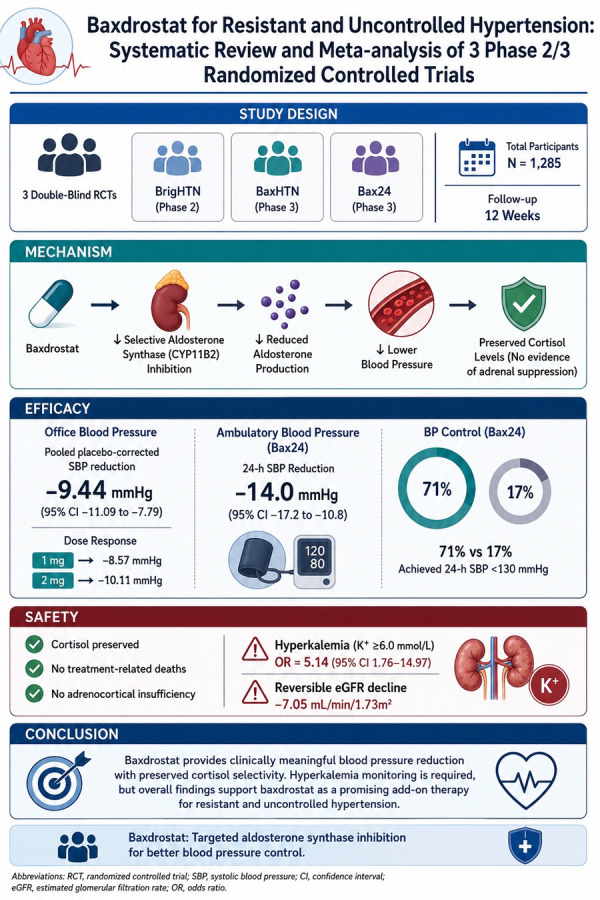

## 1 Introduction

Hypertension is the most common modifiable cause of cardiovascular death worldwide, yet many patients do not achieve target blood pressure despite multidrug therapy [1, 2, 3]. Treatment‐resistant hypertension is defined as persistently elevated blood pressure despite treatment with at least three antihypertensive agents at maximally tolerated doses, including a diuretic. This phenotype is associated with higher risks of stroke, heart failure, chronic kidney disease, and death. In these patients, excess aldosterone contributes to sodium retention, vascular remodeling, and cardiorenal injury beyond the effects of elevated blood pressure alone. Mineralocorticoid receptor antagonists are effective add‐on therapies, but their use may be limited by hyperkalemia, renal impairment, endocrine adverse effects, and compensatory increases in aldosterone [4, 5].

When aldosterone synthase (CYP11B2) is directly inhibited, it suppresses aldosterone at its production site without any disruption in cortisol synthesis [5]. Baxdrostat is an oral CYP11B2 inhibitor with approximately 100‐fold selectivity for aldosterone synthase over 11β‐hydroxylase, producing large, sustained falls in plasma aldosterone with minimal cortisol effect in phase 1 work [6]. Three trials have since tested it in clinical populations. In the phase 2 BrigHTN trial, baxdrostat reduced seated systolic BP by 8–11 mmHg versus placebo at 12 weeks in resistant hypertension, without adrenocortical insufficiency or serious drug‐related events [7]. The phase 3 BaxHTN trial confirmed a further ~9–10 mmHg reduction when added to background therapy, with hyperkalemia emerging as the primary safety signal [8, 9]. When phase 2 trial was performed in patient of CKD and uncontrolled hypertension, a similar ~8 mmHg BP reduction was observed, but hyperkalemia rates were elevated, which highlights that for how long this drug was used in nephrology practice [10].

Baxdrostat has recently been evaluated in a baxdrostat‐specific systematic review and meta‐analysis in resistant hypertension, and several class‐level syntheses have assessed aldosterone synthase inhibitors more broadly [4, 11, 12]. However, the available reviews differ from the present analysis in trial composition, outcome focus, and handling of dose‐specific estimates. In particular, the present review focuses on the three phase 2/3 double‐blind placebo‐controlled baxdrostat hypertension trials, incorporates the published Bax24 ambulatory blood pressure trial, separates clinically relevant 1 and 2 mg dose estimates, and evaluates hyperkalemia and renal function signals using absolute as well as relative measures [10, 13, 14]. This systematic review and meta‐analysis, therefore, aims to provide an updated and methodologically transparent estimate of baxdrostat efficacy and safety in resistant and uncontrolled hypertension.

## 2 Methods

### 2.1 Search Strategy and Study Selection

This systematic review and meta‐analysis were conducted in accordance with the Preferred Reporting Items for Systematic Reviews and Meta‐Analyses (PRISMA) guidelines [15]. A comprehensive search was performed in MEDLINE/PubMed, Embase, Cochrane CENTRAL, Scopus, ClinicalTrials.gov, and NEJM.org for randomized controlled trials evaluating baxdrostat for hypertension, without language restrictions, from database inception to March 2026. Search terms included “baxdrostat,” “CYP11B2 inhibitor,” “aldosterone synthase inhibitor,” “treatment‐resistant hypertension,” “uncontrolled hypertension,” “BaxHTN,” “BrigHTN,” and “Bax24.” Reference lists of included trials and relevant review articles were manually screened for additional eligible studies.

Trials were eligible for inclusion if they: (i) were randomized, double‐blind, and placebo‐controlled; (ii) enrolled adults with hypertension receiving background antihypertensive therapy; (iii) evaluated baxdrostat at any dose versus placebo; and (iv) reported change in systolic blood pressure (SBP) as a primary or secondary outcome. Trials were excluded if they were open‐label, dose‐escalation‐only phase 1 studies, non‐randomized studies, duplicate publications, or did not report sufficient aggregate outcome data to estimate placebo‐corrected blood pressure or safety outcomes.

**PRISMA 2020 Flow Diagram**


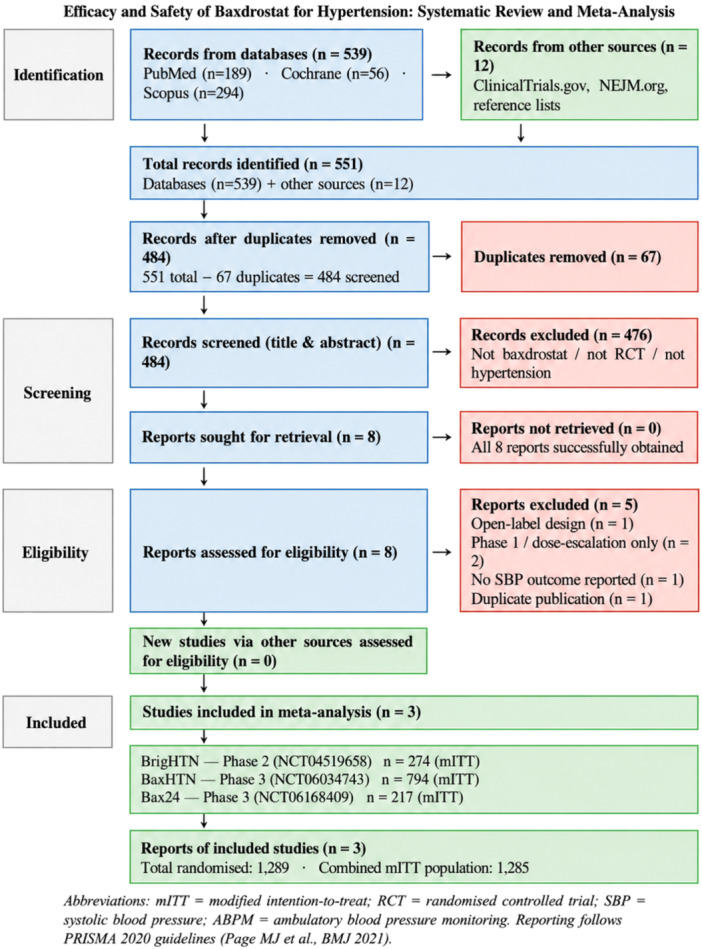


### 2.2 Included Studies

Three RCTs met eligibility criteria and were included in the quantitative synthesis: the BrigHTN Phase 2 trial (NCT04519658 [7], the BaxHTN Phase 3 trial (NCT06034743) [8], and the Bax24 Phase 3 ambulatory blood pressure monitoring trial (NCT06168409) [16]. Together, these trials enrolled 1285 participants across 214–283 clinical sites in multiple countries, with treatment durations of 12 weeks each.

### 2.3 Data Extraction

Two reviewers independently extracted data using a pre‐specified extraction form. Extracted variables included: trial name, phase, year, randomization details, hypertension type (uncontrolled or resistant), baxdrostat dose, treatment duration, sample size, baseline demographics and blood pressure, primary and secondary efficacy endpoints, pharmacodynamic outcomes (serum aldosterone, plasma renin activity, serum cortisol), and prespecified adverse events of special interest (AESI). Discrepancies were resolved by consensus with reference to the primary publications. All extracted data are presented in Table 1.

**Table 1:** Characteristics of included trials.

Characteristic	BrigHTN (Phase 2) Freeman et al. 2023	BaxHTN (Phase 3) Flack et al. 2025	Bax24 (Phase 3) Azizi et al. 2026
ClinicalTrials.gov	NCT04519658	NCT06034743	NCT06168409
Hypertension type	Treatment‐resistant	Uncontrolled + resistant	Resistant (ABPM‐confirmed)
Doses (mg)	0.5, 1, 2 once daily	1, 2 once daily	2 once daily
Primary endpoint	Change in seated SBP wk 12	Change in seated SBP wk 12	Change in 24 h ambulatory SBP wk 12
Randomization	1:1:1:1 (3 doses + placebo)	1:1:1 (2 doses + placebo)	1:1 (baxdrostat 2 mg + placebo)
Run‐in (weeks)	2 (≥ 70% adherence)	2 (≥ 80% adherence)	2 (≥ 80% + witnessed intake)
Sites/countries	USA (community + academic)	214 sites, multinational	79 sites, 22 countries
Total randomized (*N*)	275	796	218
mITT population (*N*)	274	794	217
Mean baseline seated SBP (mmHg)	147.4 ± 12.5	149.3 ± 9.3	147.6 ± 13.3
Mean baseline seated DBP (mmHg)	87.9 ± 6.7	86.5 ± 10.5	85.6 ± 10.4
Mean 24 h ABPM SBP at baseline	N/A	N/A	141.2 ± 10.1
Mean age (years)	62.3 ± 10.5	61.2 ± 11.7	60.5 ± 11.4
Male sex (%)	56	62	65
White race (%)	70	63	79
Diabetes (%)	39	38	34
Mean eGFR (mL/min/1.73 m^2^)	83.7 ± 19.5	85.0 ± 18.1	85.6 ± 18.8
Background diuretic use (%)	100	100	100
Background ACEi/ARB (%)	93	94	94
Sponsor	CinCor Pharma	AstraZeneca	AstraZeneca

*Note:* Values are mean ± SD unless otherwise specified.

Abbreviations: ABPM, ambulatory blood pressure monitoring; ACEi, angiotensin‐converting enzyme inhibitor; ARB, angiotensin‐receptor blocker; DBP, diastolic blood pressure; eGFR, estimated glomerular filtration rate; mITT, modified intention‐to‐treat; SBP, systolic blood pressure.

### 2.4 Outcomes

The primary outcome of this meta‐analysis was the placebo‐corrected change in seated office SBP from baseline to Week 12, expressed as the least‐squares (LS) mean difference (MD) with 95% confidence intervals (CI). Secondary outcomes were: placebo‐corrected change in seated office diastolic blood pressure (DBP); change in 24 h, night‐time, and daytime ambulatory SBP and DBP (from Bax24); proportion of patients achieving seated SBP below 130 mmHg; change in serum aldosterone concentration; change in serum cortisol concentration (selectivity marker); risk of potassium ≥ 6.0 mmol/L (primary safety outcome); and mean change in estimated glomerular filtration rate (eGFR). The primary meta‐analysis estimated the placebo‐corrected change in seated office SBP at Week 12 for baxdrostat doses of 1 and 2 mg. The 0.5 mg BrigHTN arm was excluded from the primary pooled efficacy estimate because it was not advanced into phase 3 and did not achieve statistical significance in the BrigHTN trial; it was retained for descriptive dose–response assessment.

### 2.5 Risk of Bias Assessment

Risk of bias was assessed independently by two reviewers using the Cochrane Risk of Bias tool version 2.0 (RoB 2) across five domains: (1) randomization process; (2) deviations from intended interventions; (3) missing outcome data; (4) measurement of outcome; and (5) selection of the reported result [17]. Each domain was judged as low risk, some concerns, or high risk. An overall judgment was assigned per study. Results of the risk of bias assessment are summarized in Table 2.

**Table 2:** Risk of bias assessment (Cochrane RoB 2.0).

Domain	BrigHTN	BaxHTN	Bax24
D1: Randomization process	Low	Low	Low
D2: Deviations from intended interventions	Low	Low	Low
D3: Missing outcome data	Low	Low	Some concerns[a]
D4: Measurement of outcome	Low	Low	Low
D5: Selection of reported results	Low	Low	Low
Overall risk of bias	Low	Low	Low

*Note:* [a] Bax24: 18 of 108 baxdrostat patients excluded from primary ABPM analysis due to missing/invalid Week‐12 measurements; no imputation performed; sensitivity analysis with multiple imputation yielded consistent results.

### 2.6 Statistical Analysis

All statistical analyses were performed in R version 4.3.1 (R Foundation for Statistical Computing, Vienna, Austria) using the metafor package (version 4.4) [18]. For the primary outcome and all continuous secondary outcomes, the pooled MD and 95% CI were estimated using a random‐effects model with restricted maximum likelihood (REML) estimation. For multi‐arm trials that contribute more than one baxdrostat dose to the same analysis, shared placebo comparators were handled to avoid double‐counting of control participants. Dose‐specific estimates were extracted separately, but the shared placebo group was either split across relevant comparisons or modeled using an approach accounting for within‐trial dependence. Primary pooled analyses were restricted to clinically relevant 1 and 2 mg doses. The 0.5 mg BrigHTN arm was summarized descriptively and included only in dose–response analyses.

Heterogeneity was quantified using the *I* ^2^ statistic and Cochran's *Q* test, with *I* ^2^ values of 25%, 50%, and 75% representing low, moderate, and high heterogeneity, respectively [19]. The between‐study variance (*τ* ^2^) was estimated using REML. A 95% prediction interval was calculated to convey the expected range of effects in a hypothetical new trial. For the binary safety outcome (potassium ≥ 6.0 mmol/L), a REML of odds ratios (ORs) on the log scale was performed; a continuity correction of 0.5 was applied to zero cells.

Pre‐specified subgroup analyses were conducted according to: (i) baxdrostat dose (1 vs. 2 mg); (ii) hypertension type (resistant hypertension only vs. mixed uncontrolled/resistant); and (iii) trial phase (Phase 2 vs. Phase 3). Subgroup differences were evaluated using a test for interaction (QM statistic). Sensitivity analyses comprised: leave‐one‐out analysis; fixed‐effects model versus REML; exclusion of Phase 2 data; and restriction to the 2 mg dose only. Formal assessment of small‐study effects or publication bias was not performed because only three trials were included, and several estimates arose from non‐independent dose arms sharing comparators. Funnel plot inspection and Egger's regression were therefore considered statistically uninformative and potentially misleading.

## 3 Results

### 3.1 Study Selection and Characteristics

Three eligible RCTs were identified and included in the meta‐analysis. Across the three trials, 1289 participants were randomized; the modified intention‐to‐treat (mITT) populations comprised 274, 794, and 217 participants in BrigHTN, BaxHTN, and Bax24, respectively. Combined mITT *N* = 1285. Baseline characteristics were well balanced across treatment arms within each trial (Table 1). The pooled trial population was predominantly male (61%–65%) and White (63%–79%), with a mean age of approximately 61 years. All patients received background diuretic therapy; 93%–95% received an angiotensin‐converting enzyme inhibitor or angiotensin‐receptor blocker; and 64%–89% received a calcium‐channel blocker. Risk of bias was low across all five RoB 2 domains in all three trials, with one domain rated “some concerns” in Bax24 owing to missing ambulatory blood pressure monitoring data at Week 12 (Table 2).

### 3.2 Primary Outcome: Seated Office SBP

**Figure 1:**
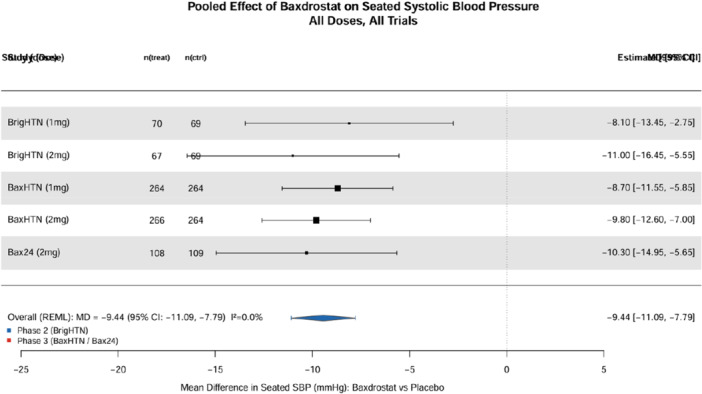
Forest plot: pooled placebo‐corrected change in seated office systolic blood pressure.

Across the clinically relevant 1 and 2 mg baxdrostat arms, the pooled placebo‐corrected reduction in seated office SBP was −9.44 mmHg (95% CI: −11.09 to −7.79; *p* < 0.001), with no observed statistical heterogeneity (*I* ^2^ = 0.0%). This estimate should therefore be interpreted as the pooled effect of the 1–2 mg dose range rather than all tested doses. The 0.5 mg BrigHTN dose was not included in the primary pooled estimate and is reported separately as part of the dose–response assessment (Figure 1).

Across individual study arms, placebo‐corrected reductions in seated SBP ranged from −8.1 mmHg (BrigHTN 1 mg; 95% CI: −13.5 to −2.8; *p* = 0.003) to −11.0 mmHg (BrigHTN 2 mg; 95% CI: −16.4 to −5.5; *p* < 0.001). In the larger Phase 3 BaxHTN trial, reductions were −8.7 mmHg (95% CI: −11.5 to −5.8; *p* < 0.0001) and −9.8 mmHg (95% CI: −12.6 to −7.0; *p* < 0.0001) for the 1 and 2 mg doses, respectively. In the Bax24 trial, where seated SBP was a secondary endpoint, the placebo‐corrected reduction was −10.3 mmHg (95% CI: −14.9 to −5.6; *p* < 0.0001). The 0.5 mg dose in BrigHTN did not achieve statistical significance (− 2.7 mmHg; *p* = 0.33), consistent with dose‐dependent pharmacology.

### 3.3 Subgroup Analysis by Dose

**Figure 2:**
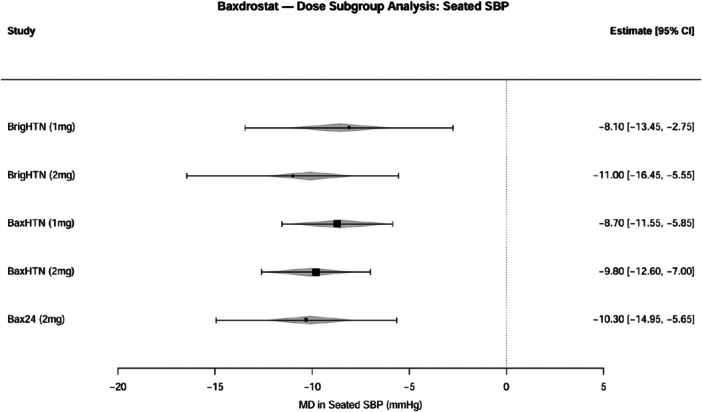
Dose subgroup analysis: seated SBP by baxdrostat dose.

Dose‐specific pooled estimates were calculated separately for baxdrostat 1 and 2 mg. The 1 mg dose was available in BrigHTN and BaxHTN, whereas the 2 mg dose was available in BrigHTN, BaxHTN, and Bax24. Therefore, the correct arm counts are k = 2 for 1 mg and k = 3 for 2 mg. Dose‐specific pooled estimates, heterogeneity statistics, and tests for subgroup differences should be recalculated using these corrected arm counts and appropriate handling of shared placebo comparators (Figure 2).

### 3.4 Subgroup Analysis: Resistant Hypertension Population

In the pre‐specified subgroup of patients with treatment‐resistant hypertension specifically—encompassing all BrigHTN participants, the BaxHTN resistant‐HTN subpopulation, and all Bax24 participants—the pooled placebo‐corrected SBP reduction was −9.61 mmHg (95% CI: −11.47 to −7.75; *I* ^2^ = 0.0%). This estimate was consistent with the primary pooled result, confirming that the blood pressure‐lowering efficacy of baxdrostat is preserved in patients meeting the strict definition of resistant hypertension (i.e., uncontrolled BP on ≥ 3 guideline‐recommended agents including a diuretic).

### 3.5 Ambulatory Blood Pressure: Bax24 Trial (Phase 3)

**Figure 3:**
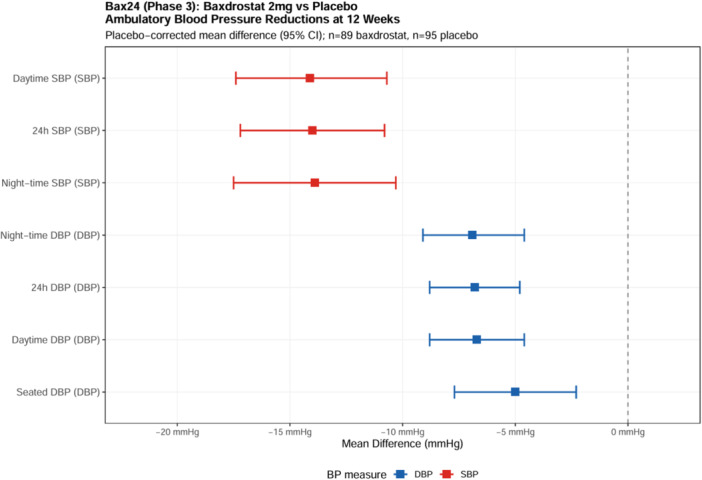
Ambulatory blood pressure outcomes: Bax24 (Phase 3).

The Bax24 trial provided the first placebo‐controlled evidence of baxdrostat's effect on 24 h ambulatory blood pressure in patients with true resistant hypertension confirmed by ambulatory BP monitoring (ABPM). At Week 12, the placebo‐corrected reductions in 24 h, daytime, and night‐time ambulatory SBP were −14.0 mmHg (95% CI: −17.2 to −10.8; *p* < 0.0001), −14.1 mmHg (95% CI: −17.4 to −10.7; *p* < 0.0001), and −13.9 mmHg (95% CI: −17.5 to −10.3; *p* < 0.0001), respectively. These reductions represent the largest placebo‐corrected ambulatory SBP reductions reported in any randomized trial in patients with true resistant hypertension to date [4]. All ambulatory DBP outcomes were similarly significant (Table 3, Figure 3).

**Table 3:** Ambulatory blood pressure outcomes from the Bax24 trial (baxdrostat 2 mg vs. placebo; *n* = 89 vs. 95).

Outcome	Baxdrostat 2 mg LS mean change (mmHg)	Placebo LS mean change (mmHg)	Placebo‐corrected MD (95% CI)	*p* value
24 h ambulatory SBP	−16.6 (−18.8 to −14.3)	−2.6 (−4.7 to −0.4)	−14.0 (−17.2 to −10.8)	< 0.0001
Night‐time ambulatory SBP	−16.0 (−18.6 to −13.4)	−2.1 (−4.6 to 0.4)	−13.9 (−17.5 to −10.3)	< 0.0001
Daytime ambulatory SBP	−16.8 (−19.2 to −14.4)	−2.7 (−5.1 to −0.4)	−14.1 (−17.4 to −10.7)	< 0.0001
Seated office SBP[a]	−14.9 (−18.2 to −11.6)	−4.7 (−7.9 to −1.4)	−10.3 (−14.9 to −5.6)	< 0.0001
24 h ambulatory DBP	−8.3 (−9.7 to −6.9)	−1.5 (−2.9 to −0.1)	−6.8 (−8.8 to −4.8)	< 0.0001
Night‐time ambulatory DBP	−7.9 (−9.6 to −6.3)	−1.1 (−2.7 to 0.5)	−6.9 (−9.1 to −4.6)	< 0.0001
Daytime ambulatory DBP	−8.4 (−9.9 to −6.9)	−1.7 (−3.2 to −0.3)	−6.7 (−8.8 to −4.6)	< 0.0001
Seated office DBP[a]	−7.6 (−9.5 to −5.7)	−2.6 (−4.5 to −0.7)	−5.0 (−7.7 to −2.3)	0.0003
24 h ABPM SBP < 130 mmHg[b]	60/85 (71%)	14/84 (17%)	OR 15.2 (6.6 to 35.2)	< 0.0001

*Note:* [a] Seated office BP was a secondary endpoint in Bax24; *n* = 108 baxdrostat, 109 placebo. [b] Only patients with baseline 24 h ABPM SBP ≥ 130 mmHg included.

Abbreviations: ABPM, ambulatory blood pressure monitoring; DBP, diastolic blood pressure; LS, least‐squares; MD, mean difference; OR, odds ratio; SBP, systolic blood pressure.

The magnitude of night‐time SBP reduction (−13.9 mmHg) was comparable to the daytime reduction (−14.1 mmHg), indicating that baxdrostat's prolonged half‐life of approximately 30 h supports effective 24 h coverage and preservation of the normal circadian BP pattern. At the same time, 71% of baxdrostat recipients versus 17% of placebo recipients achieved 24 h ABPM SBP control (< 130 mmHg) at Week 12 (OR 15.2; 95% CI: 6.6 to 35.2; *p* < 0.0001).

### 3.6 Seated DBP

The pooled placebo‐corrected reduction in seated office DBP across all meta‐analyzable study arms (BrigHTN 2 mg, BaxHTN 1 and 2 mg, Bax24 2 mg; k = 4) was −4.01 mmHg (95% CI: −5.14 to −2.88; *p* < 0.001; *I* ^2^ = 0.0%). All individual study arms demonstrated statistically significant DBP reductions, ranging from −3.3 mmHg (BaxHTN 1 mg; 95% CI: −5.2 to −1.4; *p* = 0.0008) to −5.2 mmHg (BrigHTN 2 mg; 95% CI: −8.7 to −1.6).

### 3.7 Blood Pressure Control Rates

In the BaxHTN trial, the proportion of patients achieving seated SBP < 130 mmHg at Week 12 was 39.4% with baxdrostat 1 mg and 40.0% with baxdrostat 2 mg, compared with 18.7% with placebo (OR 2.9 for both baxdrostat doses; *p* < 0.0001). BP control rates were substantially higher in Bax24, where 71% of baxdrostat 2 mg‐treated patients achieved 24 h ABPM SBP < 130 mmHg versus 17% on placebo (OR 15.2; 95% CI: 6.6 to 35.2; *p* < 0.0001). The discordance between the BaxHTN and Bax24 control rates likely reflects the more stringent ambulatory‐confirmed definition of resistant hypertension in Bax24 and the higher responsiveness of this population to aldosterone‐directed therapy.

### 3.8 Dose−Response Relationship

**Figure 4:**
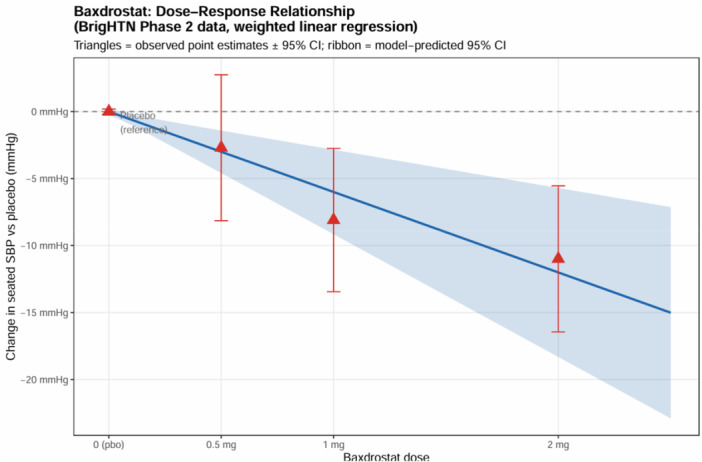
Dose–response analysis: baxdrostat dose versus placebo‐corrected seated SBP reduction (BrigHTN).

A clear dose−response relationship was observed in BrigHTN, the only trial with three active doses. Placebo‐corrected SBP reductions of −2.7, −8.1, and −11.0 mmHg were observed with 0.5, 1, and 2 mg doses, respectively. Weighted linear regression on BrigHTN dose−response data demonstrated a significant linear relationship (slope: −4.2 mmHg per mg increase; *R* ^2^ = 0.96). This dose‐dependent signal was corroborated by parallel dose‐dependent reductions in serum and urinary aldosterone concentrations (Figure 4).

### 3.9 Pharmacodynamic Outcomes

Serum cortisol concentrations were not reduced in any baxdrostat group across the three trials. In BrigHTN, the LS mean change in cortisol at the highest dose was +1.91 µg/dL (95% CI: +0.70 to +3.12). No significant differences between baxdrostat and placebo were observed for cortisol at any dose in any trial. These findings support pharmacodynamic selectivity for aldosterone synthase over 11β‐hydroxylase over 12 weeks, although longer‐term exposure data remain needed to confirm the durability of this selectivity.

Crucially, serum cortisol concentrations were not reduced in any baxdrostat group across the three trials. In BrigHTN, the LS mean change in cortisol at the highest dose (2 mg) was +1.91 µg/dL (95% CI: +0.70, +3.12), representing a slight nonspecific increase. No significant differences between baxdrostat groups and placebo were observed for cortisol at any dose in any trial. This finding definitively confirms the high selectivity of baxdrostat for aldosterone synthase (CYP11B2) relative to 11β‐hydroxylase (CYP11B1), the enzyme responsible for cortisol synthesis, which shares 93% sequence similarity with aldosterone synthase.

### 3.10 Safety Outcomes

**Figure 5:**
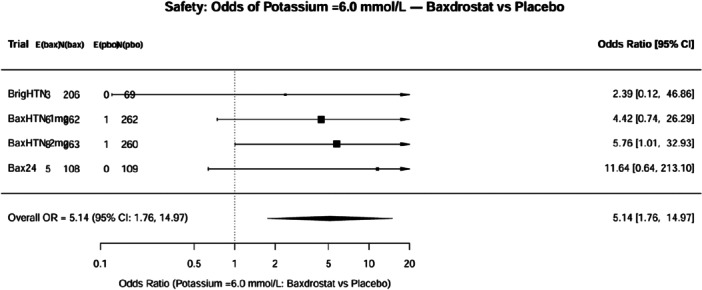
Safety forest plot: odds ratio for potassium ≥ 6.0 mmol/L (baxdrostat vs. placebo).

No deaths were attributed to baxdrostat in any trial. Rates of any adverse event were broadly comparable between baxdrostat and placebo groups: 35%–52% versus 37%–41% across trials and doses. No serious adverse events were attributed to baxdrostat by investigators in any trial. There were no cases of adrenocortical insufficiency in any participant across the three trials, consistent with the confirmed cortisol selectivity demonstrated pharmacodynamically (Figure 5).

### 3.11 Hyperkalemia

For potassium ≥ 6.0 mmol/L, absolute risks, risk differences, and relative effects were summarized. Because events were rare and zero cells occurred, ORs using a 0.5 continuity correction were considered exploratory. Sensitivity analyses using rare‐event methods, such as Mantel–Haenszel risk difference or exact/logistic approaches where feasible, were planned to assess the robustness of the safety signal.

### 3.12 Renal Function

Mean eGFR declined from baseline to Week 12 in baxdrostat‐treated patients across the Phase 3 trials: −7.0 (SD 12.8) and −6.9 (SD 12.4) mL/min/1.73 m^2^ in BaxHTN 1 mg and 2 mg groups, respectively (vs. −0.1 [SD 8.6] for placebo), and −8.7 (SD 14.0) mL/min/1.73 m^2^ in Bax24 2 mg (vs. −0.4 [SD 9.7] for placebo). The pooled mean eGFR change versus placebo was −7.05 mL/min/1.73 m^2^ (95% CI: −8.26 to −5.85; *I* ^2^ = 0.0%). In both BaxHTN and Bax24, eGFR returned toward baseline values within 2 weeks of drug discontinuation during the randomized withdrawal and follow‐up periods, confirming that the observed eGFR decline represents a reversible functional change attributable to improved blood pressure control, reducing intraglomerular perfusion pressure, rather than intrinsic nephrotoxicity.

### 3.13 Hyponatremia and Hypotension

Hyponatremia requiring clinical intervention (AESI) occurred in 0.8%–2.3% and 0.4%–1% of baxdrostat and placebo recipients, respectively, across trials. Hypotension requiring clinical intervention occurred in 1.9%–2.3% and 0.8%–1.1% of baxdrostat and placebo recipients in Phase 3. Both events were manageable, self‐limited, and did not lead to discontinuation in the majority of affected patients.

### 3.14 Sensitivity Analyses

The leave‐one‐out analysis showed no single study arm was pulling the pooled estimate in any particular direction. Across the five iterations, the MD ranged from −8.47 to −10.00 mmHg, a spread of less than 1.6 mmHg. The fixed‐effects model produced a pooled MD of −9.41 mmHg (95% CI: −10.98 to −7.84), sitting within 0.1 mmHg of the REML estimate, which suggests the pooling method choice was inconsequential here.

**Figure 6:**
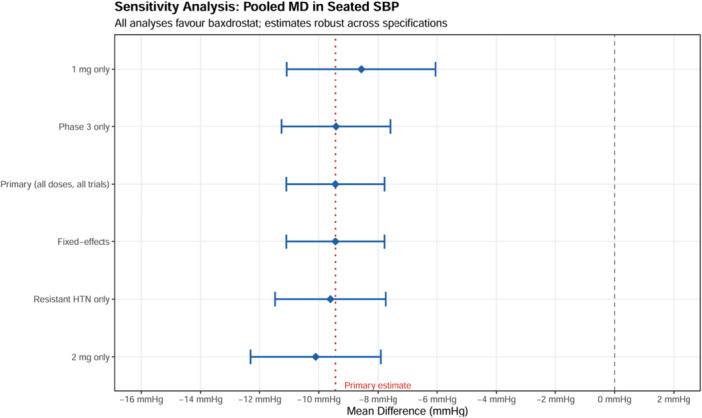
Sensitivity analysis: pooled MD in seated SBP across analytical specifications.

Narrowing the analysis to Phase 3 data only (k = 3 arms; BaxHTN 1 mg, BaxHTN 2 mg, and Bax24) gave a pooled MD of −9.42 mmHg (95% CI: −11.26 to −7.59; *I* ^2^ = 0.0%). The 2 mg dose restriction across four arms produced −10.11 mmHg (95% CI: −12.30 to −7.91; *I* ^2^ = 0.0%). Both subsets remained statistically significant, with estimates clustering tightly around the primary result (Figure 6).

### 3.15 Publication Bias

Publication bias and small‐study effects were not formally assessed because the evidence base comprised only three trials with non‐independent dose‐arm comparisons. Accordingly, no inference regarding publication bias can be made.

### 3.16 GRADE Evidence Summary

The GRADE framework was used to rate the certainty of evidence for the primary and key secondary outcomes. Certainty was rated moderate for seated office SBP reduction because the evidence came from randomized trials with low risk of bias and consistent effects, but the total number of independent trials was small, and follow‐up was limited to 12 weeks. Certainty was rated low to moderate for ambulatory BP outcomes because these were derived from a single trial. Certainty was rated moderate for seated DBP reduction and cortisol outcomes. Certainty was rated low for hyperkalemia and adrenocortical insufficiency because event counts were low, CIs were imprecise, and zero‐event data limited comparative estimation. Certainty was rated as moderate or low for eGFR change because follow‐up was short and the reversibility of the observed decline was assessed over a limited post‐treatment period. The full GRADE evidence profile is presented in Table 4.

**Table 4:** GRADE evidence summary.

Outcome	Current rating	Required revised rating
Seated SBP reduction	High	Moderate
24 h ambulatory SBP	High	Low or moderate
Night‐time SBP	High	Low or moderate
Seated DBP reduction	High	Moderate
Cortisol suppression	High	Moderate
Hyperkalemia ≥ 6.0 mmol/L	Moderate	Low
eGFR decline	High	Low or moderate
Adrenocortical insufficiency	Moderate	Low

Abbreviations: CI, confidence interval; CV, cardiovascular; DBP, diastolic blood pressure; eGFR, estimated glomerular filtration rate; GRADE, Grading of Recommendations Assessment, Development and Evaluation; MD, mean difference; OR, odds ratio; RCT, randomized controlled trial; SBP, systolic blood pressure; TRH, treatment‐resistant hypertension.

## 4 Discussion

Three phase 2/3 randomized controlled trials enrolling 1285 participants yield a pooled placebo‐corrected reduction in seated office SBP of −9.44 mmHg (95% CI: −11.09 to −7.79; *p* < 0.001; *I* ^2^ = 0.0%), with parallel pharmacodynamic evidence of sustained aldosterone suppression and preserved cortisol levels, and a statistically significant but low‐magnitude increase in clinically significant hyperkalemia (pooled OR 5.14; 95% CI: 1.76 to 14.97). The 95% prediction interval of −13.6 to −5.3 mmHg tells us that meaningful BP reduction is expected even in future populations with different baseline characteristics and trial designs. Together with the ambulatory data from Bax24, these findings position baxdrostat as an effective add‐on option for patients with resistant and uncontrolled hypertension who remain above target on existing multidrug regimens.

The ~9.4 mmHg pooled reduction here is in the same territory as recent class‐level work. Marzano et al. put placebo‐corrected SBP at 8.3 mmHg (95% CI: 5.9 to 10.7) across ASI RCTs [20], Gao et al. at 8–9 mmHg [21], and Elbahloul et al.'s network meta‐analysis placed baxdrostat 2 mg above 10 mmHg in indirect comparisons [14], which fits the dose‐specific estimate of −10.11 mmHg (95% CI: −12.30 to −7.91) we see here. What sets these three trials apart from earlier work is straightforward: the same estimate, replicated across independent data sets each with low risk of bias, in a population that was by definition already maximally treated. That is not easy to dismiss.

And it bears spelling out. Every patient enrolled was on optimized background therapy (diuretic use was universal, ACEi/ARB use ran 93%–95%) and still uncontrolled. Getting another −9.4 mmHg out of that population is a real result. For comparison, spironolactone and eplerenone as add‐on agents in resistant hypertension typically yield placebo‐corrected reductions of 4–6 mmHg. BaxHTN confirmed the BrigHTN signal almost exactly, returning −8.7 and −9.8 mmHg for 1 and 2 mg [2], with no attenuation from phase 2 to phase 3. Drug development being what it is, that consistency is not the norm, and it matters for how much weight to put on the estimate.

The Bax24 ABPM data merit particular attention. Placebo‐corrected reductions of −14.0, −14.1, and −13.9 mmHg in 24 h, daytime, and night‐time SBP are substantial for this patient group [16]. Night‐time SBP has the strongest prognostic tie to cardiovascular mortality among all ambulatory BP parameters, and here it falls as much as daytime SBP as a consequence of baxdrostat's pharmacokinetic half‐life of roughly 30 h, which keeps CYP11B2 inhibited across the full dosing interval. These ambulatory reductions also exceed the pooled 24 h SBP estimates from prior ASI meta‐analyses, which cluster around 7–10 mmHg [20, 21, 22], likely reflecting the ABPM‐confirmed resistant hypertension selection in Bax24. Rasmussen et al. arrived at the same reading in their systematic review [22].

BrigHTN is the only trial here with three active doses, and the dose−response is clean in a way that is unusual even by phase 2 standards. Placebo‐corrected seated SBP reductions of −2.7, −8.1, and −11.0 mmHg at 0.5, 1, and 2 mg, a linear regression slope of −4.2 mmHg per mg, and *R* ^2^ = 0.96 [7]. Paired with dose‐dependent reductions in serum and urinary aldosterone and compensatory plasma renin activity increases at every dose, there is no credible nonspecific explanation for the BP effect. The 0.5 mg dose did not reach statistical significance (− 2.7 mmHg; *p* = 0.33), which sets a practical floor between 0.5 and 1 mg and justifies carrying 1–2 mg forward into phase 3. In the current pooled analysis, the 1 versus 2 mg comparison did not reach statistical significance (QM = 1.93; *p* = 0.16), but the 1.35 mmHg additional reduction with the higher dose is directionally consistent with the phase 2 findings and not a number to ignore in this indication.

Taken together, the available cortisol and adrenocortical insufficiency data support baxdrostat's selectivity over 12 weeks in the studied populations. However, the current evidence remains limited to short‐term trials, and longer exposure in larger and more diverse populations will be required before definitive conclusions about long‐term adrenal safety can be made [7, 8, 16].

Clinically significant hyperkalemia, defined as potassium ≥ 6.0 mmol/L, occurred infrequently but more often with baxdrostat than placebo. In absolute terms, potassium ≥ 6.0 mmol/L occurred in approximately 2.3%–4.6% of baxdrostat‐treated participants in phase 3 trials compared with 0%–0.4% of placebo‐treated participants. The pooled OR was 5.14 (95% CI: 1.76 to 14.97), but this relative estimate should be interpreted cautiously because event counts were low and zero cells required continuity correction [23]. Absolute risks and risk differences are therefore more clinically interpretable than the OR alone [14, 24].

Two things about this signal deserve attention. First, it is heavily context‐dependent. In Dwyer et al.'s phase 2 CKD trial, any‐grade hyperkalemia hit 41% of baxdrostat recipients versus 5% with placebo [10], a gap that reflects impaired renal potassium handling in CKD and the outsized role aldosterone plays in compensating for it. The general hypertension populations in BrigHTN and BaxHTN carry nothing like that baseline risk. Second, the timing pattern is reassuring. Most hyperkalemia events appeared within the first 2 weeks and then stabilized; after drug discontinuation in both BaxHTN and Bax24, potassium returned toward baseline within 2 weeks. Front‐loaded monitoring fits better than indefinite surveillance here.

A practical protocol (baseline renal function and potassium, repeat at 2 and 4 weeks, and after any dose change) is proportionate to the risk profile and not out of step with what is already recommended for mineralocorticoid receptor antagonists. The absence of drug‐related serious adverse events or deaths across all three trials supports that calibrated approach.

The pooled eGFR decline of −7.05 mL/min/1.73 m^2^ versus placebo across BaxHTN and Bax24 is best interpreted as hemodynamic. In both phase 3 trials, eGFR returned toward baseline within 2 weeks of drug withdrawal, a trajectory inconsistent with structural nephrotoxicity. This is the same pattern seen with ACEi/ARB initiation, and in that context, it is ordinarily read as evidence of effective RAAS modulation rather than renal damage, provided the decline stabilizes. Dwyer et al. observed the same functional pattern in their CKD cohort [10].

One demographic gap runs through all three trials. Men made up 61%–65% of participants; White European participants 63%–79%. The BaxAsia trial (NCT06034745) had not reported at the time of data extraction [13] and needs to be incorporated when it does. Extrapolation to Asian populations should be treated with caution until that data is available.

### 4.1 Strengths and Limitations

All available phase 2/3 RCT evidence for baxdrostat is included here with pre‐specified outcomes and methods, and the risk of bias was consistently low across trials. GRADE certainty ratings ranged from MODERATE for seated office SBP, seated DBP, and cortisol outcomes, to LOW–MODERATE for ambulatory BP outcomes and LOW for hyperkalemia and adrenocortical insufficiency. These ratings reflect the generally low risk of bias and consistent treatment effects across trials but were downgraded because of the small number of independent studies, short follow‐up duration, reliance on a single trial for ambulatory BP outcomes, and imprecision in rare safety events. The Bax24 ABPM data set adds 24 h BP evidence that office measurements cannot provide, and the pharmacodynamic data on aldosterone, cortisol, and renin allow the clinical signal to be grounded in a plausible mechanism.

The limitations are not minor. Three trials and five study arms leave tests for heterogeneity and publication bias (Egger's test: *z* = 0.87; *p* = 0.39) underpowered; that is, a structural constraint, not a methodological choice.

Trials ran 12 weeks; there are no long‐term cardiovascular outcomes data. There is no head‐to‐head comparison with spironolactone or other mineralocorticoid receptor antagonists. Individual participant data were unavailable, which prevents exploration of effect modifiers (baseline aldosterone, background MRA use, eGFR strata) that would be genuinely useful for identifying which patients benefit most.

### 4.2 Clinical and Therapeutic Implications

What this data set supports is narrower but clinically important: baxdrostat lowers blood pressure over 12 weeks, preserves measured cortisol concentrations in the available trials, and has a safety profile that appears manageable with potassium and renal‐function monitoring. Cardiovascular outcome data and head‐to‐head comparisons with mineralocorticoid receptor antagonists are still needed to define its long‐term therapeutic role.

## 5 Conclusions

Across three randomized trials in patients with resistant or uncontrolled hypertension, baxdrostat reduced seated office SBP by approximately 9–10 mmHg and, in one trial, reduced 24 h ambulatory SBP by approximately 14 mmHg beyond placebo when added to background antihypertensive therapy. Aldosterone was reduced, and cortisol concentrations were not suppressed over 12 weeks. Hyperkalemia occurred infrequently but more often with baxdrostat than placebo, supporting the need for early potassium and renal‐function monitoring. eGFR declined modestly and appeared reversible during short‐term follow‐up, although longer‐term renal safety data are needed. Current evidence supports baxdrostat as a promising add‐on therapy, but certainty is limited by the small number of independent trials, short follow‐up, rare safety events, and absence of cardiovascular outcome data.

## Author Contributions

Ehsanullah Alokozay conceived the study, supervised the project, coordinated the research activities, and critically revised the manuscript. Rahul Falodia and Mohammad Idrees contributed to study design, literature screening, study selection, and data extraction. Darsh Paghdar performed data verification and assisted with the interpretation of the findings. Ali Msheik contributed to methodological review, data validation, and manuscript revision. Taha Alam and Saeedullah Zadran contributed to data collection, validation of extracted outcomes, interpretation of results, and critical review of the manuscript. All authors participated in drafting and revising the manuscript, approved the final version, and agree to be accountable for all aspects of the work.

## Funding

The authors have nothing to report.

## Conflicts of Interest

The authors declare no conflicts of interest.

## Data Availability

The data supporting the findings of this study are available within the article and its supporting materials. No additional data sets were generated during the current study.

## References

[1] R. M. Carey , D. A. Calhoun , G. L. Bakris , et al., “Resistant Hypertension: Detection, Evaluation, and Management: A Scientific Statement From the American Heart Association,” Hypertension 72, no. 5 (2018): e53–e90, 10.1161/HYP.0000000000000084. 30354828 PMC6530990

[2] B. Williams , G. Mancia , W. Spiering , et al., “2018 ESC/ESH Guidelines for the Management of Arterial Hypertension,” European Heart Journal 39, no. 33 (2018): 3021–3104, 10.1093/eurheartj/ehy339. 30165516

[3] P. K. Whelton , R. M. Carey , W. S. Aronow , et al., “2017 ACC/AHA/AAPA/ABC/ACPM/AGS/APhA/ASH/ASPC/NMA/PCNA Guideline for the Prevention, Detection, Evaluation, and Management of High Blood Pressure in Adults,” Hypertension 71, no. 6 (2018): e13–e115, 10.1161/HYP.0000000000000065. 29133356

[4] N. Mikhail , “Aldosterone Synthase Inhibitors for Treatment of Hypertension and Chronic Kidney Disease,” Archives of Pharmacology and Therapeutics 6 (2024): 8–12, 10.33696/pharmacol.6.048.

[5] A. F. G. Cicero , G. Tocci , A. Avagimyan , et al., “Aldosterone Synthase Inhibitors for Resistant Hypertension: Pharmacological Insights: A Systematic Review,” Drugs 85 (2025): 1429–1453, 10.1007/s40265-025-02229-2. 40885884 PMC12572095

[6] M. W. Freeman , M. Bond , B. Murphy , J. Hui , and J. Isaacsohn , “Results From a Phase 1, Randomized, Double‐Blind, Multiple Ascending Dose Study Characterizing the Pharmacokinetics and Demonstrating the Safety and Selectivity of the Aldosterone Synthase Inhibitor Baxdrostat in Healthy Volunteers,” Hypertension Research 46 (2022): 108–118, 10.1038/s41440-022-01070-4. 36266539 PMC9747611

[7] M. W. Freeman , Y. D. Halvorsen , W. Marshall , et al., “Phase 2 Trial of Baxdrostat for Treatment‐Resistant Hypertension,” New England Journal of Medicine 388 (2022): 395–405, 10.1056/nejmoa2213169. 36342143

[8] J. M. Flack , M. Azizi , J. M. Brown , et al., “Efficacy and Safety of Baxdrostat in Uncontrolled and Resistant Hypertension,” New England Journal of Medicine 393 (2025): 1363–1374, 10.1056/nejmoa2507109. 40888730 PMC7618089

[9] K. Skibicka , T. Skibicki , W. Wesołowsla , and J. Pietrzak , “New Pharmacologic Approaches in Hypertension: Baxdrostat and Zilebesiran With Insights From BaxHTN and KARDIA‐3 Trials,” Journal of Education, Health and Sport 84 (2025): 65430, 10.12775/jehs.2025.84.65430.

[10] J. P. Dwyer , N. Maklad , O. Vedin , et al., “Efficacy and Safety of Baxdrostat in Participants With CKD and Uncontrolled Hypertension: A Randomized, Double‐Blind, Placebo‐Controlled Trial,” Journal of the American Society of Nephrology 37 (2025): 299–311, 10.1681/asn.0000000849. 40913594 PMC12889919

[11] I. P. Dewi , A. M. Anggitama , E. B. Jati , and K. P. Dewi , “Comparative Efficacy and Safety of Aldosterone Synthase Inhibitors in Hypertension: A Bayesian Network Meta‐Analysis of Randomised Controlled Rials,” Open Heart 12 (2025): e003535, 10.1136/openhrt-2025-003535. 41093633 PMC12530399

[12] K. Italiya , H. A. R. Akhter , S. Khan , et al., “Abstract 4370196: Evaluating Aldosterone Synthase Inhibitors in Hypertension: A Meta‐Analysis of Efficacy, Safety, and Subgroup Outcomes Across Novel Agents,” Circulation 152 (2025): 4370196, 10.1161/circ.152.suppl_3.4370196.

[13] J. M. Flack , M. Azizi , J. M. Brown , et al., “Baxdrostat for Uncontrolled and Resistant Hypertension: Rationale and Design of the Phase 3 Clinical Trials BaxHTN, BaxAsia, and Bax24,” Hypertension Research 48 (2025): 2911–2923, 10.1038/s41440-025-02297-7. 40850955 PMC12586160

[14] M. A. Elbahloul , A. H. G. Ali , A. S. Al‐Shammari , et al., “Aldosterone Synthase Inhibitors for Resistant or Uncontrolled Hypertension: A Network Meta‐Analysis of Randomized Clinical Trials,” Journal of Hypertension 44 (2026): 542–552, 10.1097/hjh.0000000000004264. 41645642

[15] M. J. Page , J. E. McKenzie , P. M. Bossuyt , et al., “The PRISMA 2020 Statement: An Updated Guideline for Reporting Systematic Reviews,” British Medical Journal 372 (2021): n71, 10.1136/bmj.n71. 33782057 PMC8005924

[16] M. Azizi , J. M. Brown , J. P. Dwyer , et al., “Effect of Baxdrostat on Ambulatory Blood Pressure in Patients With Resistant Hypertension (Bax24): A Phase 3, Randomised, Double‐Blind, Placebo‐Controlled Trial,” Lancet 407, no. 10532 (2026): 988–999, 10.1016/S0140-6736(25)02549-8. 41794437

[17] J. A. C. Sterne , J. Savčović , M. J. Page , et al., “RoB 2: A Revised Tool for Assessing Risk of Bias in Randomised Trials,” British Medical Journal 366 (2019): l4898, 10.1136/bmj.l4898. 31462531

[18] W. Viechtbauer , “Conducting Meta‐Analyses in R With the Metafor Package,” Journal of Statistical Software 36, no. 3 (2010): 1–48, 10.18637/jss.v036.i03.

[19] J. P. T. Higgins , S. G. Thompson , J. J. Deeks , and D. G. Altman , “Measuring Inconsistency in Meta‐Analyses,” British Medical Journal 327 (2003): 557–560, 10.1136/bmj.327.7414.557. 12958120 PMC192859

[20] L. Marzano , M. Merlo , N. Martinelli , F. Pizzolo , and S. Friso , “Efficacy and Safety of Aldosterone Synthase Inhibitors for Hypertension: A Meta‐Analysis of Randomized Controlled Trials and Systematic Review,” Hypertension 82 (2025): e147–e159, 10.1161/hypertensionaha.124.23962. 39886765

[21] Y. Gao , X. Mu , and X. Pang , “Efficacy and Safety of Aldosterone Synthase Inhibitors for Uncontrolled Hypertension: A Meta‐Analysis of Randomized Controlled Trials and Systematic Review,” Frontiers in Pharmacology 16 (2025): 1664810, 10.3389/fphar.2025.1664810. 41059210 PMC12497756

[22] A. A. Rasmussen , K. L. Nordestgaard , U. Simonsen , and N. H. Buus , “Blood Pressure‐Lowering Effects of Aldosterone Synthase Inhibitors: A Systematic Review,” Basic & Clinical Pharmacology & Toxicology 137 (2025): e70080, 10.1111/bcpt.70080. 40693351 PMC12281267

[23] L. Tanriverdi , M. Dogan , A. Hernandez , et al., “Aldosterone Synthase Inhibitors Effectively Lower Blood Pressure but Increase Hyperkalemia Risk: A Meta‐Analysis of Randomized Trials,” supplement, Circulation 152, no. S3 (2025): 4348313, 10.1161/circ.152.suppl_3.4348313.

[24] X. Tu , Q. Song , Q. Tang , H. Tan , H. Zhang , and C. Fan , “Efficacy and Safety Outcomes of Aldosterone Synthase Inhibitors for Resistant Hypertension: A Meta‐Analysis of Randomized Controlled Trials,” Journal of the Renin‐Angiotensin‐Aldosterone System 27 (2026): 14703203251411193, 10.1177/14703203251411193.

